# Combined Phytochemical Sulforaphane and Dietary Fiber Inulin Contribute to the Prevention of ER-Negative Breast Cancer via PI3K/AKT/MTOR Pathway and Modulating Gut Microbial Composition

**DOI:** 10.3390/nu17122023

**Published:** 2025-06-17

**Authors:** Huixin Wu, Brittany L. Witt, William J. van der Pol, Casey D. Morrow, Lennard W. Duck, Trygve O. Tollefsbol

**Affiliations:** 1 Department of Biology, College of Arts and Sciences, University of Alabama at Birmingham, Birmingham, AL 35233, USA; huixin3@uab.edu (H.W.); bwitt95@uab.edu (B.L.W.); 2 Department of Microbiology, Heersink School of Medicine, University of Alabama at Birmingham, Birmingham, AL 35205, USA; 3 Center for Clinical and Translational Science, University of Alabama at Birmingham, Birmingham, AL 35233, USA; 4 Department of Cell, Departmental & Integrative Biology, Heersink School of Medicine, University of Alabama at Birmingham, Birmingham, AL 35294, USA; 5 Department of Med-Gastroenterology, Shelby Biomedical Research Building, University of Alabama at Birmingham, Birmingham, AL 35294, USA; 6 O’Neal Comprehensive Cancer Center, Heersink School of Medicine, University of Alabama at Birmingham, Birmingham, AL 35294, USA; 7 Integrative Center of Aging Research, University of Alabama at Birmingham, Birmingham, AL 35294, USA; 8 Nutrition Obesity Research Center, University of Alabama at Birmingham, Birmingham, AL 35294, USA; 9 Comprehensive Diabetes Center, Heersink School of Medicine, University of Alabama at Birmingham, Birmingham, AL 35294, USA; 10 University Wide Microbiome Center, University of Alabama at Birmingham, Birmingham, AL 35294, USA

**Keywords:** breast cancer, inulin, epigenetic therapy, tumor growth, gut microbiome

## Abstract

**Background:** Breast cancer (BC) is the second most common cancer among women in the United States. It has been estimated that one in eight women will be diagnosed with breast cancer in her lifetime. Various BC risk factors, such as age, physical inactivity, and smoking, play a substantial role in BC occurrence and development. Early life dietary intervention with plant-based bioactive compounds has been studied for its potential role in BC prevention. Sulforaphane (SFN), an isothiocyanate, is an antioxidant and anti-inflammatory agent extracted from broccoli sprouts (BSp) and other plants. Dietary supplementation of SFN suppresses tumor growth by inducing protective epigenetic changes and inhibiting cancer cell proliferation. Inulin, as a dietary fiber, has been studied for alleviating GI discomfort and weight loss by promoting the growth of beneficial bacteria in the gut. **Objective:** Early-life combinatorial treatment with both phytochemical SFN and potential prebiotic agent inulin at lower and safer dosages may confer more efficacious and beneficial effects in BC prevention. **Methods:** Transgenic mice representing estrogen receptor-negative BC were fed 26% (*w*/*w*) BSp and 2% (*w*/*v*) inulin supplemented in food and water, respectively. **Results:** The combinatorial treatment inhibited tumor growth, increased tumor onset latency, and synergistically reduced tumor weight. Gut microbial composition was analyzed between groups, where *Ruminococcus*, *Muribaculaceae*, and *Faecalibaculum* significantly increased, while *Blautia*, *Turicibacter*, and *Clostridium sensu stricto* 1 significantly decreased in the combinatorial group compared with the control group. Furthermore, combinatorial treatment induced a protective epigenetic effect by inhibiting histone deacetylases (HDACs) and DNA methyltransferases (DNMTs). Intermediates in the AKT/PI3K/MTOR pathway were significantly suppressed by the combinatorial treatment, including PI3K p85, p-AKT, p-PI3K p55, MTOR, and NF-κB. Cell cycle arrest and programmed cell death were induced by the combinatorial treatment via elevating the expression of cleaved-caspase 3 and 7 and inhibiting the expressions of CDK2 and CDK4, respectively. Orally administering *F. rodentium* attenuated tumor growth and induced apoptosis in a syngeneic triple-negative breast cancer (TNBC) mouse model. **Conclusions:** Overall, the findings suggest that early-life dietary combinatorial treatment contributed to BC prevention and may be a potential epigenetic therapy that serves as an adjunct to other traditional neoadjuvant therapies.

## 1. Introduction

Breast cancer (BC) is among the most diagnosed cancers and the leading cause of death worldwide from cancer in women. The occurrence of mortality of BC is continuously increasing despite the advanced screening and treatment methods [1]. Dysregulation of the expression of the estrogen receptor (ER) is found in over 60% of BC patients [2,3]. Hormone receptor-negative BC consists of 25% and triple receptor-negative BC comprises about 10% of the subtypes of BC [4]. ER-negative BC patients have fewer treatment options compared with hormone receptor-positive BC patients, and primary treatments such as locoregional and systemic therapies have significant toxicities [1,5]. Therefore, strategies that prevent BC occurrence and progression have been of great interest to the scientific community and the public society. Dietary intervention that increases the consumption of vegetables, whole grains, and fruits that are rich in fibers, vitamins, and bioactive components has been associated with reduction in cancer, diabetes mortality, and cardiovascular disease [6]. A healthful diet likely prevents BC by shaping gut microbiota, regulating metabolism in both bacterial population and the host, reducing reactive oxygen species, and strengthening host immunity. Contained in health-benefiting diets, bioactive compounds such as phytochemicals have been studied for their BC prevention effects. Sulforaphane, an isothiocyanate, is known for its anti-inflammatory, antioxidant, and anticancer properties [7]. Early-life and transgenerational dietary supplementation with sulforaphane (SFN)-enriched broccoli sprouts (BSp) has an ER-negative BC-preventive effect through inducing protective epigenetic changes in the mammary tumors in mice [8,9]. Epigenetic modifications substantially affect cancer initiation and progression, and ‘epigenetic diets’ are of great interest in promoting beneficial epigenetic modifications in the host and contributing to cancer prevention [10]. Our previous studies revealed that SFN-enriched BSp increased protective epigenetic changes, including inhibiting the expression of histone deacetylases (HDACs) and DNA methyltransferases (DNMTs) and suppressing tumor development by inhibiting cancerous cell proliferation and survival [11,12].

In addition to the phytochemicals that directly impact epigenetic modifications in mammary tumor cells, a health-benefiting diet also contains a variety of fibers and vitamins. High total fiber, including soluble and insoluble fibers, consumption is associated with reduced risk of BC [13]. The digestion and absorption of dietary fibers are mediated by millions of gut microbes. Gut microbiota are our ‘forgotten organ’ that interacts with the host immune system and regulates gut homeostasis. Dysbiosis of gut microbiota that consist of a higher population of pro-tumoral bacterial species is associated with gastrointestinal (GI) cancer and BC by increasing reactive oxygen species and the expression of inflammatory factors [14,15]. Inulin, a dietary fiber, is a commercially available dietary supplementation for weight loss and GI discomfort and can potentially restore healthy gut microbiota communities. Studies have shown that inulin can ameliorate inflammation in alcoholic liver disease [16]. Dietary supplementation of 8–15% (*w*/*v*) inulin contributed to BC prevention by modulating gut microbiota toward a short-chain fatty acid (SCFA)-producing population and inducing epigenetic changes [17]. Though daily intake of inulin has been estimated to be up to 10 g/person, and the safety of inulin and oligofructose has been evaluated by legal authorities worldwide, high doses of inulin is not suitable for long-term consumption because it can potentially increase osmotic pressure and cause intestinal discomfort that varies from person to person [18]. Therefore, it is essential to test the optimal dosages of dietary supplementation for health purposes in cancer prevention models.

Combinatorial dietary treatment with lower doses of each component offers a safer option for long-term early-life dietary intervention. This strategy is more efficacious and can synergistically prevent ER-negative BC progression, compared with conventional single-agent treatment [8,19]. Oligofructose-enriched inulin (IN) may promote the growth of healthy gut microbiota that facilitate the digestion of fibers in BSp and produce beneficial bacterial metabolites. The combination of IN and BSp can potentially synergistically induce more efficacious epigenetic modifications in mammary tumors. Gut microbiota establishment and epigenetic alterations in critical periods such as embryogenesis and postnatal early life can fundamentally affect the susceptibility of individuals to chronic diseases and cancers in later life [20]. Diet supplementation with sulforaphane-rich BSp at 26% (*w*/*w*) has been studied for its impact on epigenetic-related modifiers and gut microbial composition [21]. As a well-studied dietary fiber and potential prebiotic, 8–15% (*w*/*v*) inulin induced changes in gut microbiome and protective epigenetic modifications [17]. In this study, 2% (*w*/*v*) inulin was used because it was substantially lower than the previously studied physiologically reasonable level, 8% (*w*/*w*) [14], thus preventing possible side effects of high concentration of inulin, such as bloating or loose stool. Here, the gut microbial compositions of the control, BSp, inulin, and combination groups were investigated. We hypothesize that early-life combinatorial dietary treatment with BSp and a low dosage of inulin may modify the gut microbial composition, bring protective epigenetic changes, and suppress tumor cell proliferation in the ER-negative preclinical BC mouse model.

## 2. Materials and Methods

### 2.1. Animal Experiments—Mouse Model

The transgenic mouse model FVB/N-Tg (MMTV-Erbb2) NK1Mul/J (Her2/neu) was purchased from the Jackson Laboratory (Bar Harbor, ME, USA). Female mice in this model spontaneously developed ER-negative mammary tumors, with a median tumor onset at 20 weeks of age due to the overexpression of the activated oncogene *Erbb2* [9,11]. The ER-negative EO771 syngeneic mouse model (associated with luminal B tumor subtype) was generated by injecting 2 × 10^5^ cells under the mammary fat pad of each mouse.

Mice were housed in the Animal Resource Facility, and IACUC Protocol 10033 was approved by the Animal Resources Program at the University of Alabama at Birmingham (UAB). The mice were maintained under controlled conditions: 24 ± 2 °C, 40–60% humidity, and a 12 h dark/light cycle.

The effective sample size for the mouse experiments was determined by a one-sided two-proportion comparison using a power calculator (https://powerandsamplesize.com/Calculators/ (accessed on 1 January 2020)). The effective size was calculated to achieve 80% power for detecting the effect of the combinatorial dietary treatment on BC prevention or the effect of *F. rodentium* oral administration on BC prevention at α = 0.05. A total of 10 mice per group were used for the combinatorial dietary treatment experiment. A total of 15 mice per group were used for the bacteria-administered experiment.

### 2.2. Dietary Treatment

The BSp diet was customized with 26% (*w*/*w*) BSp (Natural Sprout Co., Springfield, MO, USA) supplemented to AIN-93G diet pellets (TestDiet, Richmond, IN, USA). Detailed ingredients are provided in previous publications [9,12] and are provided in Supplemental Materials labeled Supplemental Table S14 The amount of BSp consumed by a mouse is equivalent to a daily intake of 234 g of BSp for an adult [12]. A 2% (*w*/*v*) Orafti^®^ Synergy1, oligofructose-enriched inulin (Beneo, Parsippany, NJ, USA), was supplemented in the drinking water (https://www.beneo.com/human-nutrition/human-nutrition-products/functional-fibres (accessed on 1 January 2020)). The dosage of inulin used in this experiment was lower than that used in a previous study, which suggested that 8% (*w*/*v*) inulin, equivalent to 40 g of fiber per day, is physiologically achievable and practical for human patients [22]. Forty female Her2/neu mice, aged 4 weeks, were randomly divided into 4 groups (10 mice/group): (1) control group: AIN-93G diet; (2) BSp group: BSp diet as described above; (3) IN group: inulin in drinking water as described above; and (4) combination group: combination of the BSp diet and inulin in water. Clustering randomization was performed for each cage, and the double-blind method was used to avoid bias during formal analysis [23]. Mice were provided food and water ad libitum. Food and water intake were measured at 15 and 25 weeks of age for the control and all treatment groups. Body weight was measured every week before tumor development and every 2 weeks after tumor development (Figure S2). Treatment started at 4 weeks of age and continued until 29 weeks of age, when all mice in the control group developed tumors with an average volume greater than 1.0 cm^3^.

### 2.3. Mammary Tumor Growth Evaluation

Tumor incidence, tumor volume, and the latency of tumor development were measured and recorded weekly. Tumor volumes (cm^3^) were calculated using the formula length (cm) × width (cm) × (height (cm)) × 0.523 [8,24]. For the mice that were developing tumors, each mouse was monitored at least twice weekly for signs of distress and was euthanized if they appeared moribund after consultation with a veterinarian. Body condition score was used to monitor these animals 3 times a week; the mice were euthanized if their conditions decreased to a score of 2 or less. If the tumor burden in the animal exceeded 10% of the total body weight, the mouse was euthanized. Mice were sacrificed with carbon dioxide from a gas cylinder with a flow rate set to displace 30–70% of the chamber/cage volume/min, and the mammary tumors were collected, weighed, and stored in liquid nitrogen for subsequent analysis. Animal procedures and related experiments were reviewed and approved by the UAB Institutional Animal Care and Use Committee.

### 2.4. Cell Culture

The EO771 cell line, obtained from Dr. Lalita Samant’s laboratory at the University of Alabama at Birmingham, passed the rodent/mouse pathogen testing. EO771 cells were cultured in Dulbecco’s Modified Eagle’s Medium (DMEM, Corning, Somerville, MA, USA) supplemented with 10% Fetal Bovine Serum and 1% penicillin/streptomycin. The cells were incubated in a 5% carbon dioxide incubator at 37 °C with controlled humidity.

### 2.5. Bacterial Strains and Animal Treatment

Freeze-dried *Faecalibaculum rodentium* was purchased from the Leibniz Institute DSMZ–German Collection of Microorganisms and Cell Culture GmbH (Braunschweig, Germany). *F. rodentium* was cultured in pre-reduced Chopped Meat Medium (Anaerobe Systems) and incubated in an anaerobic chamber (gas atmosphere N_2_/CO_2_/H_2_, 90:5:5).

*F. rodentium* was grown to an optical density (O.D.) of 600 nm ≅ 0.6, corresponding to approximately 5 × 10^7^ UFC/mL. A 200 µL dose of *F. rodentium* culture was administered by oral gavage to each mouse every other day throughout the study for 4 weeks.

### 2.6. Gut Microbiome Analysis

Fecal samples (6–9 samples/group) were collected from the control group and all treated groups of the Her2/neu mouse model at two time points: (1) 15 weeks of age, before tumor onset, and (2) 25 weeks of age, after tumor onset. Genomic DNA was extracted from approximately 50 mg of fecal specimens/sample using the Fecal DNA Isolation Kit (Zymo Research, Irvine, CA, USA), following the manufacturer’s instructions. The extracted DNA was immediately used for PCR or stored in Tris-EDTA buffer (pH 8) at 4 °C. The sample DNA concentration was quantified using a microspectrophotometer (Thermo Fisher, Waltham, MA, USA) [25].

The amplicon library was generated by PCR amplification of the V4 region of the 16S rRNA gene with uniquely barcoded primers [8]. The oligonucleotide primers used for PCR amplification were as follows (Eurofins Genomics, Inc., Huntsville, AL, USA):

- Forward V4: 5′ AATGATACGGCGACCACCGAGATCTACACTATGGTAATTGTGTGCCAG CMGCCGCGGTAA-3′;
- Reverse V4: 5′ CAAGAGAAGACGGCATACGAGATNNNNNNAGTCAGTCAGCCGGACTA CHVGGGTWTCTAAT-3′.

The quantification of samples was performed using the PicoGreen dsDNA Reagent (Thermo Fisher Scientific, Waltham, MA, USA). PCR products were visualized on a UV illuminator after agarose gel electrophoresis and extracted from the gel using the QIAquick Gel Extraction Kit (Qiagen, Germantown, MD, USA). The sequencing of approximately 250 bp paired-end reads of the V4 region of the 16S rRNA gene was carried out by NextGen sequencing on the Illumina MiSeq platform (Illumima, San Diego, CA, USA) [17,26].

The generated FASTQ files were assessed for quality control using FastQC and library construction. The processed library was used for Quantitative Insight into Microbial Ecology (QIIME) analysis [27]. DADA2, a pipeline that filters out low abundance sequences due to polymerase chain reaction or sequencing errors, was used to cluster sequences into amplicon sequence variants (ASV) and assign taxonomies [28,29]. Multiple sequence alignment of ASVs was generated by PyNAST [29,30]. Alpha diversity was calculated with observed species (measuring richness only), PD whole tree (including phylogeny), Shannon, and Simpson indices. Beta diversity was calculated using the Bray–Curtis and weighted UniFrac methods to quantify dissimilarity between treatment groups (BSp, IN, and Combination) and the control group [17,24].

### 2.7. Western Blotting Analysis

Three randomly selected tumor samples per group from the Her2/neu mice were used for Western blotting. Protein samples were prepared by homogenizing 50–100 mg of tumor tissues in the T-PER Tissue Protein Extraction Reagent (Thermo Fisher Scientific) with a protease/phosphatase inhibitor cocktail (100×) (Cell Signaling, Danvers, MA, USA). The samples were centrifuged at 10,000× *g* at 4 °C for 5 min. Protein concentration was determined using the Bradford Assay.

NuPAGE Tris-HCl gels (Invitrogen, Carlsbad, CA, USA), 4–15%, were used to separate denatured protein samples by electrophoresis. After transferring the protein samples to nitrocellulose membranes, several antibodies were used to probe the targets, including Hdac1, Hdac2, Hdac3, Hdac4, Hdac6, Hdac8, Dnmt1, Dnmt3a, Dnmt3b, p53, PTEN, Rb, p-Rb, PI3K p85, p-Akt S473, p-Akt T308, MDM2, NF-κB p65, mTOR, p-PI3K p55, pro-caspase-3, cleaved-caspase-3, pro-caspase-7, cleaved-caspase-7, pro-caspase-9, Bcl-2, Bcl-xL, Cdk1, Cdk2, Cdk4, Cyclin A, Cyclin D1, and Cyclin E1. A detailed list of antibodies is provided in the Supplementary Material (Supplementary Table S11).

Clarity Max Western ECL Blotting Substrates (Bio-Rad, Hercules, CA, USA) were applied to the membranes, and protein bands were visualized using the ChemiDoc XRS+ system (Bio-Rad). ImageJ version 1.54 g was used to quantify protein expression. B-actin was probed on the same membrane as a loading control for quantification purposes. Detailed information of antibodies can be found in Supplementary Table S12.

### 2.8. Statistical Analysis

Data were analyzed using GraphPad Prism (version 10.0.0 (131)). Tumor incidence was analyzed by the chi-square test. For tumor weight, tumor latency, and protein expression, comparisons between two groups were analyzed using a two-tailed Student’s *t*-test; comparisons among four groups were analyzed using one-way ANOVA, followed by Dunnett’s multiple comparisons test. Error bars are presented as means ± SEMs. Statistical differences between groups were considered significant at *p* < 0.05.

Synergistic, additive, and antagonist effects were analyzed using Drug Plates analysis (https://sicodea.shinyapps.io/shiny/ (accessed on 8 June 2025))

## 3. Results

### 3.1. Combinatorial Dietary Supplementation of BSp and Inulin Was Effective in Preventing ER-Negative Mammary Tumorigenesis in Her2/neu Mice

**Figure 1:**
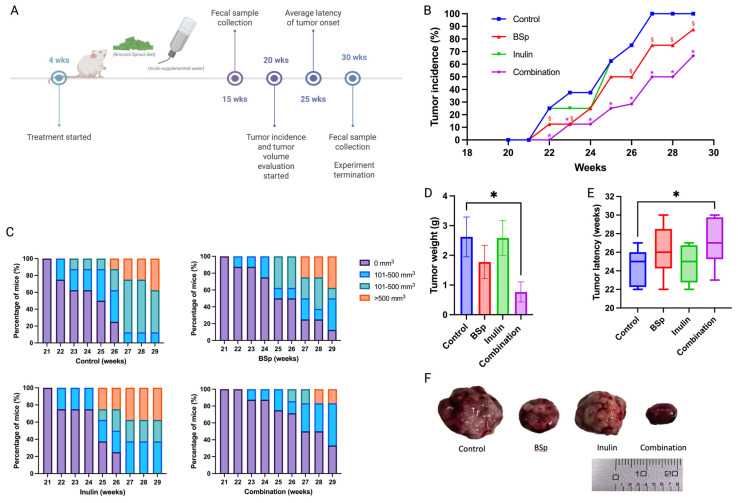
Combinatorial dietary administration of BSp and/or inulin suppressed the mammary tumor development in Her2/neu mice. (**A**) Schematic representation of the experimental design. Dietary treatment initiated from weaning to the termination point of this experiment. (**B**) Tumor incidence, (**C**) tumor volume, (**D**) tumor weight (measured at the termination point), (**E**) tumor latency, and (**F**) representative images of the excised tumors recorded at the termination point. Columns, mean; Bars, SEM; *, *p* < 0.05, combinatorial group §, *p* < 0.05, BSP group. n = 10.

A Her2/neu transgenic mouse model was employed because this model spontaneously develops Erbb2-overexpressed ER-negative mammary tumors suitable for studying human BC [31]. The BSp treatment concentration was determined by previous publications, whereby 26% (*w*/*w*) BSp diet induced a positive effect on BC prevention in the same mouse model [8], and the concentration of inulin, 2% (*w*/*v*), was modified based on previous studies [17]. This study explored the cancer prevention effect of a combination of 26% (*w*/*w*) BSp in diet and 2% (*w*/*v*) inulin in water in the transgenic mouse model Her2/neu. The experimental setup and sample collection time points are illustrated (Figure 1A). Tumor incidence and tumor volume were measured weekly. The combinatorial dietary BSp and/or inulin administration significantly decreased tumor incidence from the tumor onset in the combinatorial group to the termination point of this study. The tumor incidence of the BSp group was significantly lower at all measurement points except for 24 and 25 weeks. Inulin alone did not have a protective effect on tumor growth; however, the combinatorial group significantly and synergistically decreased tumor incidence at all measured time points (Figure 1B). Furthermore, the combinatorial diet treatment greatly decreased tumor volume over the observation period (Figure 1C). Notably, the combinatorial diet treatment group had synergism and significantly decreased tumor weight and increased tumor latency compared with the control group (Figure 1D,E). Synergism, antagonism, and additive effects were evaluated (Table S12).

### 3.2. The Impact of Combinatorial Dietary Supplementation on Gut Microbial Composition Before and After the Onset of Tumor

The average tumor onset of the Her2/neu transgenic mouse model was 20 weeks of age. The changes in gut microbial compositions were evaluated at two time points: before the tumor onset at 15 weeks of age and after the tumor onset at 25 weeks of age (Figure 1A).

#### 3.2.1. The Impact of Combinatorial Diet on Gut Microbial Diversity and Composition Before the Onset of Tumor

**Figure 2:**
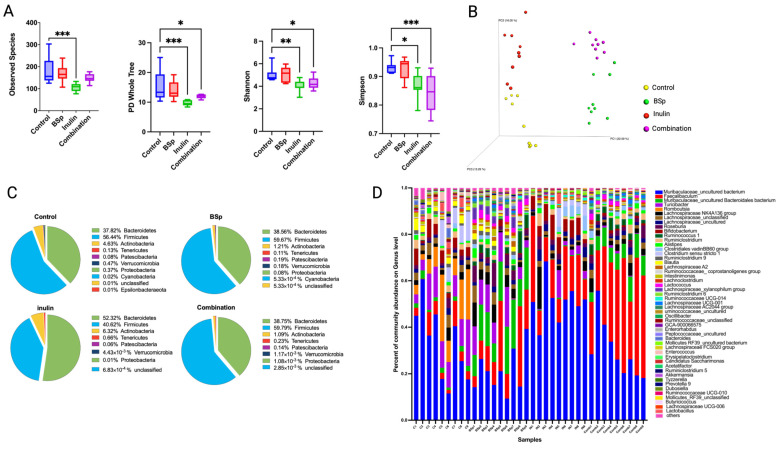
Gut microbial composition changes before the tumor onset. Distinct differences in microbial diversity and composition between the treatment groups and the control group were shown by (**A**) alpha diversity: observed species, PD whole tree, Shannon diversity, and Simpson diversity; (**B**) Bray–Curtis beta diversity PCoA plot; (**C**) top 10 most abundant phylum level changes between groups; and (**D**) top 50 most abundant genera-level changes among groups. Column, mean; error bars, SEM; *, *p* < 0.05; **, *p* < 0.01; ***, *p* < 0.001, significantly different from the control group. n = 9.

The Her2/neu transgenic female mice spontaneously developed ER-negative mammary tumor with a mean onset of 20 weeks of age [31]. Fecal samples were collected from mice at 15 weeks of age to study the impact of dietary treatment on gut microbiome. Observed species, PD whole tree, Shannon, and Simpson diversity showed significant lower diversity of gut microbial composition in the combinatorial group compared with the control group, indicating that the gut microbiome was shaped toward defined populations by the dietary treatment before the tumor onset (Figure 2A). The Bray–Curtis beta diversity displayed in the Principal Coordinates Analysis (PCoA) plot showed distinct clustering of individual biological replicates in each group (Figure 2B). A PERMANOVA test on the Bray–Curtis and unweighted UniFrac of each treatment group compared with the control group further validated the clustering effect (Table 1). The top 10 abundant phyla of all groups are plotted in the pie charts, and the top 50 abundant genera of all groups are displayed in the bar plots (Figure 2C,D). At the phylum level, the relative abundance of Bacteroidetes increased, while the relative abundance of Firmicutes decreased in the inulin group. The relative abundance of Bacteroidetes and Firmicutes was at approximately the same level among the control, BSp, and combinatorial groups. At the genus level, the bar plots indicated the relative abundance of *Faecalibaculum* was sustainably higher in the combinatorial group compared with the control group (*p* < 0.005). Overall, the BSp component in the dietary treatment contributed a greater proportion to the alteration of gut microbial composition in the combinatorial group. BSp-fed and combination-fed groups had distinct clustering as compared with the inulin-fed and control dietary mice.

Comparison of the relative abundance of the bacterial taxonomic units among different treatment groups was analyzed using the Kruskal–Wallis test. The statistically significant differences of relative abundance of microbes between groups were determined by one-way analysis of variance, followed by false discovery rate (FDR) correction. Compared with the control group, the BSp, inulin, and combinatorial groups had numerous bacterial taxa identified at the genus and species levels. The BSp diet significantly increased the relative abundance of *Ruminococcus 1*, *Clostridium sp. Clone-44*, *Streptococcaceae*, *Muribaculaceae*, *Lactococcus lactis*, *Lachnospiraceae AC2044*, and *Lachnospiraceae UCG-001*, while it decreased the relative abundance of *Peptostreptococcaceae*, *Romboutsia*, and *Ruminoclostridium* (Supplementary Table S1). The inulin group showed a significant increase in *Lachnospiraceae* and *Faecalibaculum* and a decrease in *Blautia*, *Romboutsia*, *Ruminiclostridium 9*, and *Turicibacter* (Supplementary Table S2). The microbial compositions between the control and the combinatorial group had greater differences. The combinatorial treatment significantly increased the relative abundance of *Lachnospiraceae AC2044*, *Clostridium sp. Culture-27*, *Ruminococcus*, *Lactococcus lactis*, *Faecalibaculum*, and *Oscillibacter*, while it decreased *Enterococcus durans*, *Lachnospiraceae NK4A136 group*, *Lachnospiraceae UCG-001*, *Lachnospiraceae FCS020*, *Romboutsia*, *Blautia*, and *Turicibacter*. Notably, the relative abundance of *Faecalibaculum* increased from 4.7% to 35% by the combinatorial treatment (Supplementary Table S3). Overall, the changes of gut microbial composition in the combinatorial group were shaped by BSp diet and inulin-supplemented water towards higher proportions of health-benefiting bacteria before the tumor onset.

#### 3.2.2. The Impact of Combinatorial Diet on Gut Microbial Diversity and Composition After the Onset of Tumor

**Figure 3:**
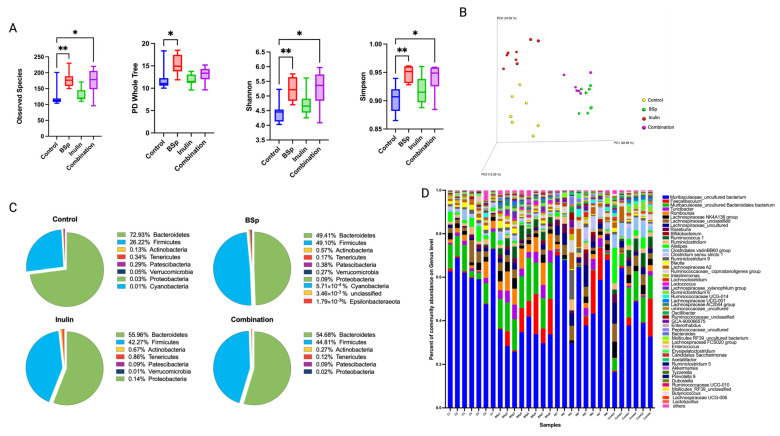
Gut microbial composition changes after the tumor onset. Distinct differences in microbial diversity and compositions between the treatment groups and the control group are shown by (**A**) alpha diversity: observed species, PD whole tree, Shannon diversity, and Simpson diversity; (**B**) Bray–Curtis beta diversity PCoA plot; (**C**) top 10 most abundant phylum level changes between groups; and (**D**) top 50 most abundance genera level changes among groups. Column, mean; error bars, SEM; *, *p* < 0.05; **, *p* < 0.01, significantly different from the control group. n = 6–8.

The dietary treatment was conducted throughout the experiment. Gut microbial composition changes over time along with the tumor development. Therefore, the gut microbial composition after the tumor onset was important to better understand the relationship between gut microbes and cancer progression. Fecal samples were collected from mice at 25 weeks of age before the termination of the experiment. The alpha diversity of all groups showed significant increases in observed species, Shannon diversity, and Simpson diversity of the combinatorial group compared with pre-treatment or compared with the control arm. The BSp group had significant increases in observed species, PD whole tree, Shannon diversity, and Simpson diversity (Figure 3A). The result indicated that the diversity of gut microbes increased over time as the dietary treatment continued. BSp and combinatorial groups had noticeably higher alpha diversity compared with the control group and the inulin-fed group. Beta diversity revealed a distinct clustering of microbial communities in each treatment group compared with the control group (Figure 3B). The significance of clustering was determined by a PERMANOVA test (Table 2). The BSp group and the combination group shared relatively less differentiated gut microbial composition than the control or inulin group (Figure 3B). Compared with inulin-supplemented water, BSp diet had a considerably higher impact on shaping the gut microbiome of the combinatorial group. The top 10 abundant phyla and the top 50 abundant genera of all groups are represented in pie charts and bar plot, respectively (Figure 3C,D). The BSp group had the highest Firmicutes-to-Bacteroidetes ratio, followed by the combinatorial group. Furthermore, the top 50 abundant genera plot showed a higher diversity but less defined gut microbial composition in the combinatorial group compared with the control group, and these changes may be contributed by the persistent dietary treatment. Overall, our findings suggest that continuous dietary treatment led to an increase in the diversity of gut microbial composition even during the development of mammary tumors.

The relative abundance of bacterial taxonomic units of all treatment groups was analyzed using the Kruskal–Wallis test, followed by the FDR correction. Interestingly, the significantly different microbial communities identified in the BSp group (n = 19 at the species level) were markedly higher than that identified in the inulin group (n = 1 at the family level) and the combinatorial group. The BSp group showed a significant increase in *Lachnospiraceae AC2044*, *Lachnospiraceae UCG-001*, *Clostridium sp. Clone-44*, *Ruminococcus*, *Muribaculaceae*, *Turicibacter*, and *Rumonococcaceae* and a decrease in *Mollicutes RF39* (Supplementary Table S4). The inulin group significantly decreased *Clostridiales Family XIII* (Supplementary Table S5). The combinatorial groups had no significantly different bacterial taxonomic unit identified (Supplementary Table S6). Our findings suggest that the BSp diet continued to shape gut microbiota during the development of mammary tumors, while inulin-supplemented water had a weaker impact. Longitudinal changes of gut microbial composition within each treatment group over time were also investigated to study the influence of treatment period and tumor development on the microbial communities within groups. The relative abundance of bacterial composition within groups was compared between two time points, 15 and 25 weeks of age that corresponded to before and after the tumor onset, respectively. The control group showed a significant decrease in *Bifidobacteriaceae*, *Erysipelotrichaceae*, and *Lactobacillaceae* after the tumor onset (Supplementary Table S7). *Clostridiaceae 1* and *Peptostreptococcaceae* increased, while *Streptococcaceae* decreased in the BSp group (Supplementary Table S8). The inulin group and the combinatorial group had several significantly changed microbial communities identified at the phylum and order levels (Supplementary Tables S9 and S10). The plasma SCFA profile was measured by LC–MS/MS. Changes in SCFA family members were identified in the treatment groups compared with the control group but were not significant (Supplementary Data). Overall, the relative abundance of gut microbes did not largely change before and after tumor development for all dietary treatment groups, though the control group had greater alterations. These results indicate that the treatments had a constant impact on gut microbiota throughout the experiment; therefore, their microbial composition was maintained before and after tumor development.

### 3.3. Combinatorial Dietary BSp and/or Inulin Administration Changed the Expression of Epigenetic-Related Proteins

**Figure 4:**
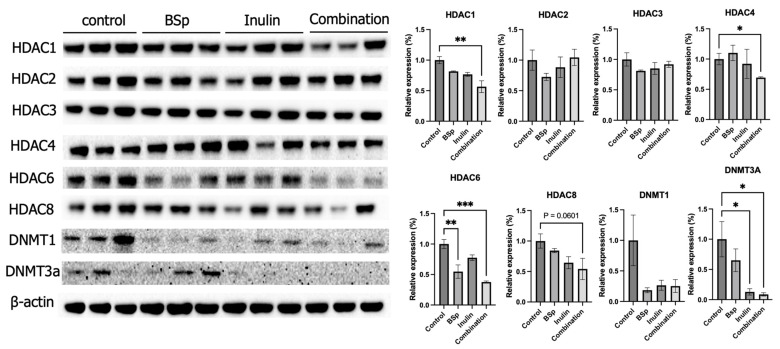
Protein expression of key epigenetic modification-related factors in the mammary tumors from the BSp, inulin, combination, and control groups. Western blotting was performed to quantify the protein expressions of Hdac1, Hdac2, Hdac3, Hdac4, Hdac6, Hdac8, Dnmt3a, and Dnmt1 in the mammary tumor samples of the dietary treatment groups and the control group. β-actin was used as the loading control. Column, mean; error bars, SEM; *, *p* < 0.05; **, *p* < 0.01; ***, *p* < 0.001, significantly different from the control group. n = 3, biological replicates.

Our previous research suggested that early-life dietary intervention with combinatorial phytochemicals such as BSp and green tea polyphenols or bioactive plant-extracted compounds such as inulin increased the SCFAs levels in the plasma of the same transgenic mouse model [17,21]. SCFAs are histone deacetylase (HDAC) inhibitors and potential DNA methyltransferase (DNMT) inhibitors [17,32]. Therefore, the protein expression of HDACs and DNMTs was evaluated in this study for exploring the direct influence of a combination of BSp and inulin on mammary tumors. The protein expressions of key epigenetic-modulatory enzymes HDACs, including HDAC1, HDAC2, HDAC3, HDAC4, HDAC6, and HDAC8, and DNMTs, including DNMT1 and DNMT3a, were analyzed. Synergism, antagonism, and additive effects were evaluated (Table S11). The results show that the combinatorial treatment significantly decreased the expressions of HDAC1, HDAC4, HDAC6, and DNMT3a (Figure 4). Furthermore, the combinatorial group synergistically decreased the expressions of HDAC1, HDAC4, HDAC6, and HDAC8. HDAC1 (*p* < 0.01), HDAC4 (*p* < 0.05), HDAC6 (*p* < 0.01 and 0.001), and HDAC8 (*p* = 0.0601) depicted synergistic effects when treated with BSp and IN in combination. DNMT3A (*p* < 0.05) and DNMT1 illustrated an additive effect when analyzed. HDAC2 and HDAC3 had an antagonistic effect when treated with BSp and IN. The synergistic effect seen within the Class I and II HDACs demonstrates the ability for these targets to remodel chromatin and reactivate transcription. These findings suggest that combinatorial, not single, administration of dietary compounds was efficient to induce protective epigenetic changes in mammary tumors.

### 3.4. Combinatorial Dietary BSp and/or Inulin Administration Changed the Expression of Intermediates in the PI3K/AKT/mTOR Pathway

**Figure 5:**
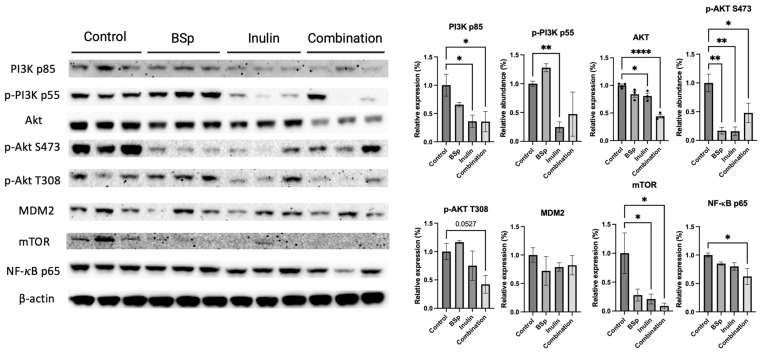
Protein expression of intermediates in the PI3K/AKT/mTOR pathway in the mammary tumors from the BSp, inulin, combination, and control groups. Western blotting was performed to quantify the protein expressions of PI3K p85, p-PI3K p55, AKT, p-AKT S473, p-AKT T308, MDM2, mTOR, and NF-*κ*B in the mammary tumor samples of dietary treatment groups and the control group. β-actin was used as the loading control. Column, mean; error bars, SEM; *, *p* < 0.05; **, *p* < 0.01, **** *p* < 0.0001, significantly different from the control group. n = 3, biological replicates.

The phenotypic data showed that the combinatorial dietary treatment was more efficient on BC prevention. Average tumor weight and tumor incidence were decreased. We hypothesized that the combinatorial treatment suppressed tumor growth by inhibiting fundamental biological processes and signaling pathways involved in cell growth. The PI3K/AKT/mTOR pathway regulates cell proliferation and survival [33]. Therefore, the protein expression of key intermediates in the PI3K/AKT/mTOR pathway was measured (Figure 5). The expressions of PI3K p85, AKT, p-AKT S473, p-AKT T308 (*p* = 0.0527), mTOR, and NF-*κ*B significantly decreased in the combinatorial group. Synergism, antagonism, and additive effects were evaluated (Table S11). Synergism analysis revealed that AKT, p-AKT T308, and NF-*κ*B were synergistically inhibited by combinatorial treatment. Meanwhile, only p-AKT S473 significantly decreased in the BSp group, and PI3K p85, p-PI3K p55, p-AKT S473, and mTOR decreased in the inulin group. AKT (*p* < 0.05 and 0.0001), NF-κB (*p* < 0.05), and p-AKT T308 (*p* = 0.0527) depicted synergistic effects when treated with the combinatorial group. PI3K p85 (*p* < 0.01) and mTOR (*p* < 0.05) illustrated an additive effect when analyzed. P-AKT S473 (*p* < 0.05, 0.01, and 0.001) and p-PI3K p55 (*p* < 0.01) had an antagonistic effect when treated with BSp and IN. The compilation of inhibition reduced survival signals and aligns with the phenotypic data in Figure 1, as well as the patterns seen in Figure 5. The results suggest that the combinatorial treatment induced a greater cell-growth-inhibiting effect than the single treatment compared with the control group.

### 3.5. Combinatorial Dietary BSp and/or Inulin Administration Changed the Expression of Apoptosis-Related Proteins

**Figure 6:**
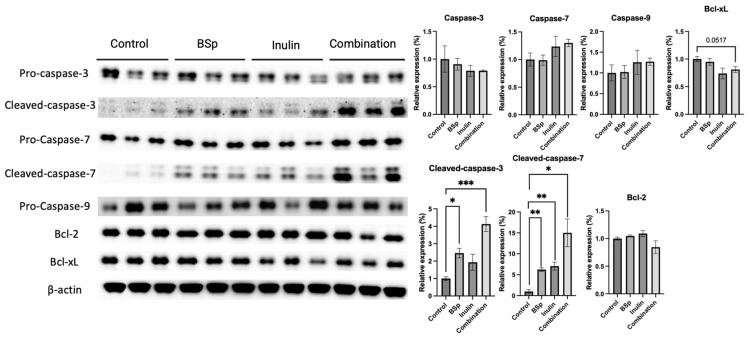
Protein expression of key factors in the intrinsic apoptosis pathway in the mammary tumors from the BSp, inulin, combination, and control groups. Western blotting was performed to quantify the protein expression of pro-caspase-3, cleaved caspase-3, pro-caspase-7, cleaved-caspase-7, pro-caspase-9, Bcl-2, and Bcl-xL in the mammary tumor samples of the dietary treatment groups and the control group. β-actin was used as the loading control. Column, mean; error bars, SEM; *, *p* < 0.05; **, *p* < 0.01, ***, *p* < 0.001, significantly different from the control group. n = 3, biological replicates.

Apoptosis, programmed cell death, and a controlled process of the cell machinery closely regulate cell survival and death [34]. The protein expressions of intrinsic apoptosis-related proteins such as pro-caspases and caspases were measured in the mammary tumors (Figure 6). Synergism, antagonism, and additive effects were evaluated (Table S11). The expression of pro-caspases, including caspase 3, caspase 7, and caspase 9, were not significantly changed by the treatment groups. The expressions of cleaved-caspase 3 and cleaved-caspase 7 were significantly and markedly induced by the combinatorial treatment. In addition, the expression of cleaved-caspase 3 was significantly increased in the BSp group, and the expression of cleaved-caspase 7 was induced in the BSp and inulin groups. The expression of Bcl-xL was notably decreased in the combinatorial group (*p* = 0.0517). Among measured candidates, cleaved-caspase-3 (*p* < 0.05 and 0.001), caspase-7 (*p* < 0.05 and 0.01), and Bcl-2 were synergistically inhibited in the combinatorial group. An increase in both cleaved caspase protein levels, seen in Figure 6, indicates apoptosis since both targets are mediators in programmed cell death. The induction of cleaved caspases involved in the intrinsic apoptosis pathway indicates that the combinatorial treatment may suppress tumor growth through increased programmed cell death.

### 3.6. Combinatorial Dietary BSp and/or Inulin Administration Changed the Expression of Intermediates in the Cell Cycle Pathway

**Figure 7:**
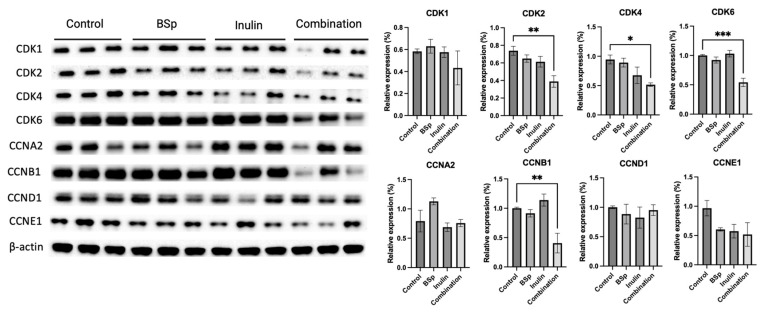
Protein expression of intermediates in the cell cycle regulation in the mammary tumors from the BSp, inulin, combination, and control groups. Western blotting was performed to quantify the protein expressions of CDK1, CDK2, CDK4, CDK6, CCNA2, CCNB1, CCND1, and CCNE1 in the mammary tumor samples of the dietary treatment groups and the control group. β-actin was used as the loading control. Column, mean; error bars, SEM; *, *p* < 0.05; **, *p* < 0.01, *** *p* < 0.001 significantly different from the control group. n = 3, biological replicates.

The previous data supported our hypothesis that the combinatorial dietary treatment contributes to cancer prevention via suppressing the growth of mammary tumor cells and inducing apoptosis. The cell cycle is among other essential biological processes that regulate cell proliferation. Therefore, the expression of cell cycle pathway-related intermediates was evaluated (Figure 7). Synergism, antagonism, and additive effects were evaluated (Table S11). The combinatorial group synergistically and significantly suppressed the expressions of CDK2 (*p* < 0.01), CDK4 (*p* < 0.05), CDK6 (*p* < 0.001), and CCNB1 (*p* < 0.01). A single administration of BSp or inulin was not sufficient to induce significant changes in cell cycle-related protein expression. These CDKs and cyclin B can be seen as protumorigenic when hyperactive, so a visual decrease in protein expression in Figure 7 supports the intended mechanism of action by dietary compounds. Our findings indicate that the combinatorial treatment is more efficient in promoting cell cycle arrest in the mammary tumor cells.

### 3.7. Combinatorial Dietary BSp and/or Inulin Administration Induced the Expression of Tumor Suppressors

**Figure 8:**
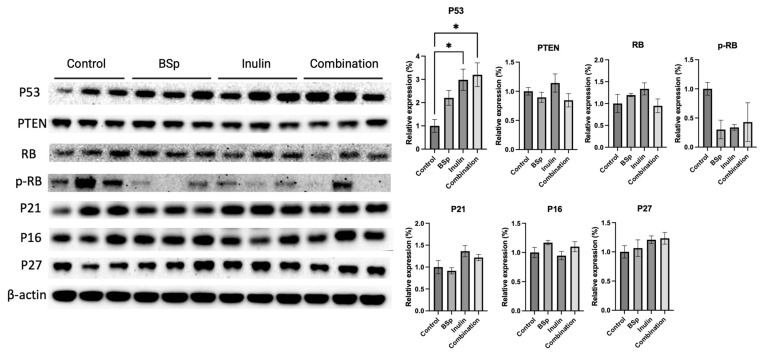
Protein expression of intermediates in the cell cycle regulation in the mammary tumors from the BSp, inulin, combination, and control groups. Western blotting was performed to quantify the protein expressions of P53, PTEN, Rb, p-RB, P21, P16, and P27 in the mammary tumor samples of the dietary treatment groups and the control group. β-actin was used as the loading control. Column, mean; error bars, SEM; *, *p* < 0.05; significantly different from the control group. n = 3, biological replicates.

During the tumor development, the expression of oncogenes and tumor suppressors is critical for regulating cell growth. Tumor suppressors function against the abnormal proliferation and tumorigenesis. Therefore, the tumor suppressors P53, phosphatase, and tensin homolog deleted on chromosome 10 (PTEN), Rb, P21, P27, and P16 were measured in the mammary tumors (Figure 8). Synergism, antagonism, and additive effects were evaluated (Table S11). The results show that P53 is greatly and significantly increased in the combinatorial group and the inulin group. P21 and P27 increased in the treatment groups compared with the control group, though not significantly. No significant changes were observed for PTEN or Rb across different treatment groups and compared with the control group.

### 3.8. Faecalibaculum Rodentium Delayed ER–Negative Breast Tumor Development in Mice

**Figure 9:**
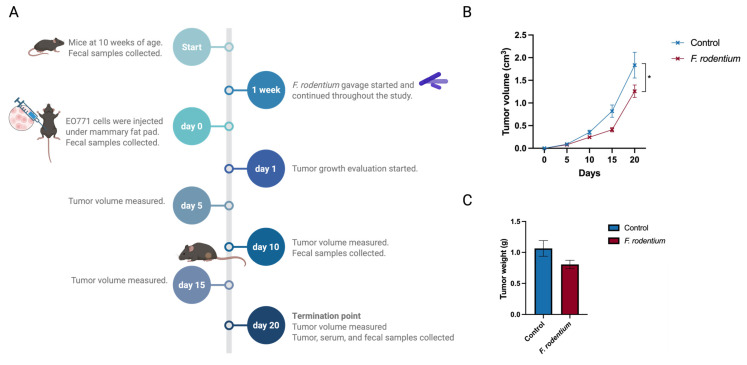
Oral administration of *Faecalibaculum rodentium* delayed the mammary tumor development in an EO771 syngeneic mouse model: (**A**) Schematic representation of the experimental design. (**B**) Tumor volume. (**C**) Tumor weight was evaluated. Columns, mean; bars, SEM; *, *p* < 0.05. n = 15.

A remarkable increase in the *Faecalibaculum* genus was found in the combinatorial dietary-treated group compared with the control group before the onset of tumor. *Faecalibaculum rodentium*, the only identified species under the genus, is known as a propionate producer. Therefore, we studied the potential cancer prevention role of *F. rodentium* in an ER-negative syngeneic mouse model. The experimental design and sample collection time points are illustrated (Figure 9A). *F. rodentium* was administrated by oral gavage every other day throughout the study. Fecal samples were collected to analyze gut microbial composition and to confirm the relative abundance of *Faecalibaculum* colonization (Figure S1). A syngeneic mouse model was created by injecting a murine-originated ER-negative cell line, derived spontaneously from C57BL/6 mice, under mammary fat pads of mice. Tumor volume was measured every 5 days from the injection time point. Tumor volume significantly decreased in the *F. rodentium*-treated group compared with the control group (Figure 9B). Tumor weight greatly decreased in the *F. rodentium* treated group, though not significantly (Figure 9C). Our data indicate that an increased relative abundance *of F. rodentium* in gut microbiota was associated with attenuated cancer development in this ER-negative syngeneic mouse model.

### 3.9. F. rodentium Administration Changed the Expressions of Apoptosis-Related Proteins and Key Epigenetic Regulators HDACs

**Figure 10:**
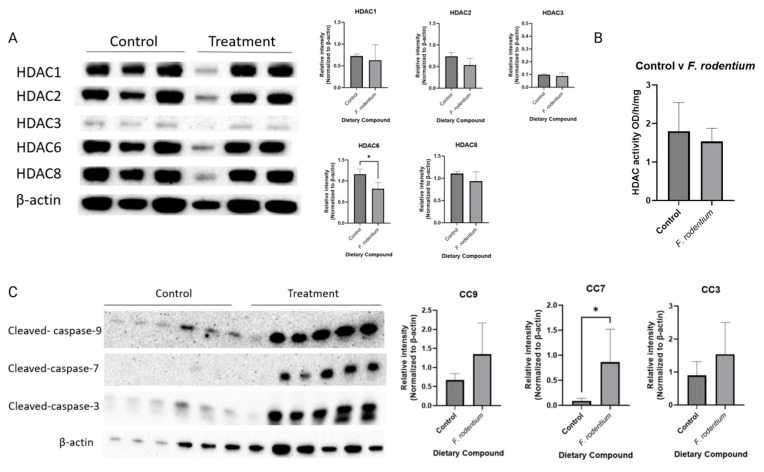
Protein expression of key epigenetic regulators HDACs and key factors in the intrinsic apoptosis pathway in the mammary tumors from the *F. rodentium* and control groups. Western blotting was performed to quantify the protein expressions of (**A**) HDAC1, HDAC2, HDAC3, HDAC6, and HDAC8, n = 3. (**B**) cleaved-caspase-3, cleaved caspase-7, and cleaved-caspase-9 in the mammary tumor samples. β-actin was used as the loading control. (**C**) Cleaved-caspase-9, cleaved-caspase-7, and cleaved-caspase-3, n = 6. Column, mean; error bars, SEM; *, *p* < 0.05, significantly different from the control group. n = 3 or 6, biological replicates.

Combinatorial dietary treatment of broccoli sprout and inulin induced key factors in the apoptosis pathway and suppressed the expression of the epigenetic-related proteins HDACs and DNMTs (Figure 4 and Figure 6). Therefore, the protein expressions of cleaved-caspases and HDACs were measured in tumor samples. *F. rodentium* administration significantly decreased the expression of HDAC6, and greatly decreased the expression of other members in the HDAC family (Figure 10A). HDAC enzyme activity decreased in the *F. rodentium* group compared with the control group, though not significantly (Figure 10B). The protein expressions of cleaved caspases that regulate the apoptosis pathway were measured in tumor samples. The expressions of cleaved-caspase-3, cleaved-caspase-7, and cleaved-caspase-9 remarkably and significantly increased in the *F. rodentium*-treated group compared with the control group.

## 4. Discussion

Dietary intervention in early life is a feasible and safe strategy for preventing breast cancer (BC). Traditional therapies, such as chemotherapy and hormone therapy, tend to benefit individuals with overexpressed hormone receptors. However, estrogen receptor-negative (ER-negative) BC patients have more limited treatment options. Therefore, incorporating dietary interventions with phytochemicals and other beneficial bioactive components is crucial for reducing BC incidence and improving the overall quality of life for BC patients.

Our previous studies have shown that early-life and transgenerational supplementation of broccoli sprout (BSp) suppressed ER-negative BC development by inhibiting cell proliferation and survival, and inducing epigenetic modulation both in vitro and in vivo [9,11]. The intake of sulforaphane (SFN), a key compound in BSp, depends on the amount of BSp consumed daily. It has been estimated that four cups of broccoli per person per day are sufficient to elicit protective epigenetic changes [11]. In vitro, the synergistic anticancer effects of BSp were observed when combined with other bioactive compounds, such as genistein, epigallocatechin gallate (EGCG), and withaferin A [35,36]. In vivo studies have shown that a BSp-supplemented diet, combined with other bioactive compounds like green tea polyphenols, synergistically prevents BC progression in ER-negative transgenic mouse models [12].

The efficacy of dietary treatments is partially mediated by the gut microbiota, which plays a crucial role in digestion, absorption, and the secretion of gut bacterial byproducts that interact with the host. Thus, the role of gut microbiota in phytochemical-based dietary treatments is significant. Long-term BSp treatment has been shown to alter the gut microbial composition, increasing the relative abundance of *Akkermansia muciniphila* and the *Lachnospiraceae* family, while decreasing *Lactococcus* abundance [21]. Since gut microbiota are closely associated with the anticancer efficacy of bioactive diets, we included a potential prebiotic component in our study to combine with the well-studied BSp diet. The plant-based prebiotic inulin is a commercially available dietary supplement that promotes bowel movement and may replenish beneficial gut bacterial populations [17]. This dietary fiber can only be digested by gut microbes, making it an excellent nutrient source for beneficial bacterial communities that are promoted by BSp.

Our study investigated the effect and mechanism of combining BSp and inulin (IN) in an ER-negative transgenic mouse model (Her2/neu). The combination of BSp and a low concentration 2% (*w*/*v*) of inulin was administered after the Her2/neu mice were weaned, and the treatment continued throughout the experiment. The anticancer effects of the treatment groups were evaluated based on tumor volume, tumor incidence, tumor weight, and tumor latency. The combinatorial treatment significantly reduced tumor volume, decreased tumor incidence at all measured time points, and was the only treatment group that significantly reduced tumor weight while increasing tumor latency. These results suggest that the combination of BSp and IN greatly contributed to the suppression of BC compared with single-compound treatments. We did not see any significant difference between the observed parameters of the groups, indicating that water/food/fluctuation in weight was not attributable to our discussed results. Importantly, the combination group exhibited a synergistic impact on tumor incidence for all weeks except 23, as Supplementary Table S12 depicts, and was significantly different from the control group in Figure 1D. The tumor latency also depicts a significant difference from the control group in Figure 1E, supporting the genuine treatment effect rather than an indirect consequence of weight loss from food intake.

BSp is known to alter the gut microbial composition. In a previous study, we found that dietary supplementation with inulin increased the populations of beneficial bacteria, such as *Lactobacillus murinus*, *Faecalibacterium*, and *Akkermansia*, in the Her2/neu transgenic mouse model [17]. Gut microbial communities can metabolize both soluble and insoluble carbohydrates to generate energy, release gas, and secrete byproducts [37]. In the present study, the gut microbiomes of all treatment groups were analyzed using 16S rRNA sequencing. Before tumor onset, the alpha diversity of the combinatorial treatment group was lower compared with that of the control group, indicating that the gut microbiota of the treated group were shaped toward a less diverse but more defined population. The beta diversity of the gut microbiome between groups showed distinct and significant clustering, with each group harboring its own distinct bacterial population as a result of dietary treatment. The top 10 most abundant phyla in all groups before tumor onset exhibited similar abundance across the control, BSp, and combination groups. The inulin (IN) group, however, showed notable differences in the abundance of *Firmicutes* and *Bacteroidetes*, suggesting that inulin supplementation distinctly altered the gut microbiota.

After tumor onset, the alpha diversity of the combinatorial treatment group significantly increased compared with the control group. The beta diversity and clustering effect remained significant but became less distinct after tumor onset, which is consistent with our previous findings [21,38]. At the phylum level, the microbial compositions in the BSp, IN, and combination groups were more similar to each other than to the control group. This result suggests that the efficacy of dietary interventions on gut microbiota is persistent, and long-term dietary treatments continuously shape microbial composition toward increased diversity, even during tumor development.

Analysis of microbial composition revealed significant differences between groups, with distinct microbial communities identified before and after tumor onset. Before tumor onset, several bacterial genera and species showed significant differences in relative abundance between groups. Notably, several cancer-associated bacterial genera and species were identified in the taxonomy studies of each treatment group. In the BSp group, the relative abundance of *Ruminococcus* 1, *Lactococcus lactis*, and the *Lachnospiraceae* family increased, while the relative abundance of *Peptostreptococcaceae* decreased. *Ruminococcus* has been isolated from the milk of healthy individuals, and the population of *Ruminococcus gnavus* is reduced in gastrointestinal cancers [39,40]. Several strains of *Lactococcus lactis*, such as *L. lactis* NK34 and *L. lactis* KC24, have probiotic properties and exhibited strong cytotoxic effects when co-cultured with the BC cell line MCF-7 [41,42]. The *Lachnospiraceae* family contains numerous anaerobic bacterial strains that produce butyrate and other short-chain fatty acids (SCFAs), which help to inhibit intestinal inflammation and maintain intestinal homeostasis [43]. The genus *Peptostreptococcus*, particularly *P. anaerobius*, has been shown to promote colorectal carcinogenesis and regulate tumor immunity in colorectal cancer [44].

In the inulin treatment group, the relative abundance of *Lachnospiraceae* and *Faecalibaculum* significantly increased, while the abundance of *Turicibacter* decreased. *Faecalibaculum rodentium* and its human homolog, *H. biformis*, are SCFA producers and are under-represented in tumorigenesis [25,45]. Moreover, *Faecalibaculum rodentium* and its metabolic products significantly reduced tumor growth in an AOM/DSS-induced colorectal cancer mouse model [25]. Therefore, the human homolog of this anti-tumorigenic strain could potentially have therapeutic effects for BC patients.

*Turicibacter* was found to be significantly enriched in patients with constipation and was abundantly detected in AOM-DSS-treated colorectal cancer mice [46,47]. In our study, compared with the single-treatment groups, the combinatorial group had a greater impact on the composition of the microbiota than the individual agents. Several bacterial taxa identified in the combinatorial group were similar to those in the BSp- and inulin-singly treated groups, including *Lachnospiraceae*, *Ruminococcus*, *L. lactis*, *Faecalibaculum*, and *Turicibacter*. Interestingly, *Faecalibaculum* was the most prominently altered genus among all microbes identified in the taxonomy studies. The abundance of *Faecalibaculum* increased from 4.7% in the control group to 35% in the combinatorial group. Given the anticancer effects of *Faecalibaculum rodentium*, it is important to further explore its mechanism in BC initiation and progression.

Additionally, the combinatorial group harbored several other cancer-related microbes. *Oscillibacter* was found at higher levels in mucosal colorectal microbiota from colorectal cancer patients. *Oscillibacter* was also shown to promote the differentiation of regulatory T cells (Tr1) in the gut and was associated with inhibited pro-inflammatory Th17 responses in hepatocellular carcinoma [48,49].

Notably, after tumor onset, the taxonomy analysis in our study revealed few statistically significant differences in bacterial genera or species between the treatment groups. The BSp treatment continued to shape the gut microbial communities, inducing significant changes in the gut microbiome. However, the inulin and combinatorial groups showed little to no difference in microbial composition compared with the control group, suggesting that while BSp consistently contributed to alterations in gut microbial composition, the inulin and combinatorial treatments had a lesser impact on gut microbes during tumor development. Interestingly, the potential anticancer effect was conferred by health-benefiting bacteria before tumor onset, but tumor development counteracted the changes in gut microbial composition induced by the single and combinatorial treatments.

Furthermore, longitudinal comparisons within each treatment group were made before and after tumor onset. More pronounced differences between groups were observed before tumor development, which is consistent with the possibility that the altered microbiota partially mediate the protective effect of combinatorial treatment. Greater changes in gut microbial composition were found within the BSp group over time compared with the inulin and combinatorial groups, suggesting that the influence of BSp treatment was more susceptible to systemic changes induced by tumor development, whereas the effects of inulin and combinatorial treatments persisted throughout the experiment.

To further explore the direct anticancer efficacy of BSp, IN, and combinatorial treatments on tumorigenesis, tumor samples were collected at the experiment’s termination, and the expression of key proteins was measured. The gut microbiome analysis previously identified numerous health-benefiting bacteria, including SCFA producers. Previous studies have demonstrated that SCFAs, such as butyrate and propionate, exhibit anticancer properties in both ER-positive and ER-negative BC cell lines by inhibiting cell proliferation and migration. SCFAs can also modify histone acetylation status [50,51]. Histone deacetylation, regulated by histone deacetylases (HDACs), leads to decreased expression of tumor suppression genes involved in cell cycle regulation, cell proliferation and survival, and angiogenesis [52,53]. DNA methyltransferases, by inducing hypermethylation, silence growth-regulatory genes, contributing to uncontrolled cancer cell growth [54]. In this study, the expressions of key epigenetic regulators, HDACs and DNMTs, were measured. The combinatorial treatment significantly decreased the expressions of HDAC1, HDAC4, HDAC6, and DNMT3a. BSp and inulin treatments individually suppressed the expressions of HDAC6 and DNMT3a, respectively. These results suggest that the combination of BSp and IN is more efficacious in eliciting protective epigenetic changes in the mammary tumors of Her2/neu mice. Specific molecular mechanisms that could elucidate the synergistic capabilities of BSp and inulin include the epigenetic mechanism of HDAC inhibition from inulin that activates Nrf2 (also activated by BSp) [12]. The capabilities of Nrf2 activation caused by HDAC inhibition produces multiple downstream effects such as inhibiting NF-kB, based on the collaboration of both BSp and inulin [55].

Epigenetic modifications directly regulate the activation and suppression of critical genes for tumor cell proliferation and survival. The PI3K/AKT/mTOR signaling pathway, one of the most commonly altered pathways driving BC progression, has been targeted in numerous preclinical BC therapies [33,56,57]. The PI3K/AKT signaling cascade also plays a significant role in endocrine resistance by promoting the proliferation, metabolism, and survival of BC tumor cells [58]. Downstream intermediates of the PI3K/AKT pathway, such as NF-kB, a proinflammatory transcription factor, promote invasive BC tumor development. Inhibiting NF-kB is associated with higher disease-free survival in BC patients [59]. We evaluated the protein expression of PI3K, phosphor PI3K, AKT, phosphor-AKT, mTOR, MDM2, and the downstream factor NF-kB. The combinatorial treatment significantly decreased the expressions of PI3K p85, AKT, p AKT S473, mTOR, and NF-kB, and greatly reduced the expression of p-AKT T308. Although BSp and IN treatments showed inhibitory effects on PI3K and AKT, the combinatorial treatment exhibited a stronger overall repressive effect on intermediates of this signaling cascade. Clinically approved PI3K and mTOR inhibitors, such as Buparlisib, Pictilisib, and Alpelisib, are used for treating cancers, and more inhibitors targeting AKT and dual PI3K/mTOR inhibitors are under clinical trials [33]. Our combinatorial bioactive compounds may offer a relatively non-toxic and safer option for long-term early-life BC prevention.

The PI3K/AKT/mTOR pathway has been extensively studied due to its role in regulating cell growth, apoptosis, the cell cycle, protein synthesis, inflammatory response, and survival genes [60]. In this study, we explored the apoptosis and cell cycle signaling pathways that may be regulated by BSp and IN combinatorial treatment due to its inhibitory effect on the PI3K/AKT/mTOR signaling cascade. Deregulation of the cell cycle leads to uncontrolled proliferation of cancer cells. CDK4/6 inhibitors block the G1 to S phase transition in cancer cells, and selective CDK4/6 inhibitors such as Palbociclib and Ribociclib have been approved by EMA and FDA for treating ER-negative metastatic BC [61,62]. In our study, we found that only the combinatorial treatment significantly inhibited the expressions of CDK2, CDK4, CDK6, and CCNB1 compared with the control, suggesting that combinatorial treatment is more effective in suppressing the cell cycle. These plant-based, naturally derived cell cycle inhibitors are promising for BC prevention.

Additionally, the apoptosis pathway was investigated. Previous studies have shown that bioactive compounds such as curcumin and apigenin can repress the growth of MDA MB-231, MCF-7, and MDA-MB-453 cell lines by upregulating cleaved-caspase-3 and Bax and downregulating Bcl-2 and Bcl-xL [34]. Transgenerational long-term dietary treatment with the bioactive compound genistein increased the expression of cleaved caspase-3 and induced apoptosis, which contributed to its anticancer effect [63]. Clinically, higher expression of cleaved-caspase-3 predicts a better chemotherapy response but a worse prognosis. Low levels of cleaved-caspase-7 are associated with an unfavorable outcome in all BC types [64,65]. Moreover, activation of the intrinsic apoptotic pathway enhances the anti-estrogen response in luminal BCs [66]. In the present study, we observed significant and substantial decreases in cleaved-caspases-3 and 7 and a notable decrease in Bcl-xL (*p* = 0.0517) compared with the control group. These results indicate that programmed cell death, via the intrinsic apoptosis pathway, was induced by combinatorial treatment.

Tumor suppressors inhibit cell transformation, and their inactivation is commonly due to intragenic mutations, chromosomal deletions, or transcriptional silencing [67]. The inhibition of tumor suppressors facilitates tumor cell growth. In this study, the expression of tumor suppressors was measured in mammary tumors from Her2/neu mice. P53 expression significantly increased in the combinatorial group, and other tumor suppressors such as P16, P21, and P27 showed increases, though not statistically significant. Moreover, downregulation of P53 is associated with poor prognosis [67]. Thus, the increase in P53 expression observed in this study suggests that combinatorial treatment significantly induced tumor suppressors, which contributed to BC prevention.

The relative abundance of the genus *Faecalibaculum* greatly increased in the combinatorial treatment group compared with the control group. A previous study showed that the anti-tumorigenic strain *Faecalibaculum rodentium* contributed to reduced tumor growth in colorectal cancer models, and its metabolic product, SCFAs, controlled protein acetylation and tumor cell proliferation by inhibiting calcineurin/NFATc3 activation [25]. Therefore, we evaluated the potential cancer prevention effect of *F. rodentium* in the EO771 syngeneic mouse model. Our results showed that *F. rodentium* delayed tumor development, which was associated with induced apoptosis and increased protective epigenetic changes in mammary tumors.

## 5. Conclusions

**Figure 11:**
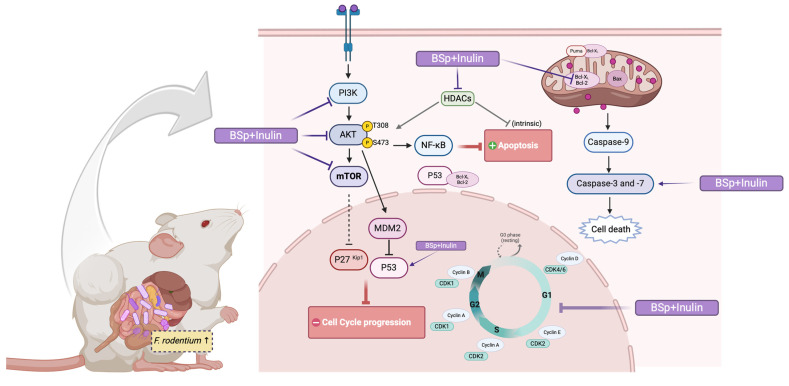
Hypothetical schematic representation of the mechanism of action for BC prevention in combinatorial diet-fed mice. A proposed mechanism illustrating the anticancer effect of the BSp and IN combinatorial dietary intervention. The combinatorial treatment induced changes in gut microbial composition, enhanced protective epigenetic modifications, inhibited the PI3K/AKT/mTOR and cell cycle signaling pathways, and promoted intrinsic apoptosis in the mammary tumors of the ER-negative BC mouse model. Upward arrows indicate activation, dashed arrows indicate indirect effects, and T-bar arrows indicate inhibition.

In summary, the BSp and IN combinatorial dietary treatment can profoundly suppress ER-negative mammary tumorigenesis by shaping gut microbial composition, inducing protective epigenetic modifications, suppressing the PI3K/AKT/mTOR signaling pathway, downregulating the cell cycle, and inducing apoptosis in the ER-negative transgenic mouse model. Furthermore, the cancer prevention effect of the combinatorial treatment may partially depend on the altered gut microbial composition, particularly the increased relative abundance of the *Faecalibaculum* genus. Since *F. rodentium* treatment suppressed tumor development, increased intrinsic apoptosis, and promoted protective epigenetic changes in the ER-negative syngeneic mouse model, this effect underscores the importance of gut microbes in BC prevention. A hypothetical schematic representation of the proposed mechanism is shown (Figure 11). A potential limitation would be the lack of validation of protein expression utilizing qRT-PCR. Additionally, there was a strenuous amount of work performed on Western blotting, which is a semi-quantitative and time-consuming technique; however, we have displayed strong data. The purpose of this additional study was to understand the impact F. rod had on the syngeneic mouse model and breast cancer progression. Additional qRT-PCR may have added a transcriptional layer to better understand the molecular changes of genes that may be influencing our findings, alongside the protein level changes we analyzed. The utilization of qPCR would have been an additional attribute that would have been more specific to quantifying the bacterial species, such as *F. rodentium*, in fecal samples from the study compared with 16S rRNA gene sequencing. Not only did the intake of *F. rodentium* increase the abundance, but the intake of BSp and IN may have created a favorable condition for *F. rodentium* to thrive in the gut, to promote the growth of all other Faecalibaculum species. An additional investigation of species-specific quantification of *F. rodentium* and other Faecalibaculum species after treatment could further clarify the connection between the combination treatment and the amount of Faecalibaculum. Polymicrobial interaction in BC occurrence and development can be evaluated by delivering mouse models with multiple strains of bacteria that are known to associate with BC. For the in vivo studies, investigating the metabolites such as folate, bile acid, and SCFA level in a plasma sample is also important to study how single- or poly-bacteria species affect BC development. This study provides a foundation for new perspectives on combinatorial dietary interventions with plant-extracted bioactive compounds. As PI3K/AKT/mTOR inhibitors, CDK4/6 inhibitors, and potential prebiotic functioning compounds, BSp and IN in combination exhibited anti-tumorigenic properties in early-life and long-term dietary interventions. Administration of dietary BSp and IN could serve as a new approach for preventing ER-negative BC. Further research into the relationship between individual gut microbes and BC progression, as well as the anticancer effects of combinatorial botanical compounds, is needed to better interpret and design dietary supplementation and neoadjuvant drugs for BC prevention and treatment.

## Figures and Tables

**Table 1:** Bray–Curtis and unweighted UniFrac of each treatment group and the control group before the onset of tumor.

	Bray–Curtis (*p*-Value)	Unweighted UniFrac (*p*-Value)
**BSp and control groups**	0.001	0.004
**Inulin and control groups**	0.001	0.001
**Combinatorial and control groups**	0.001	0.001

**Table 2:** Bray–Curtis and unweighted UniFrac of each treatment group and the control group after the onset of tumor.

	Bray–Curtis (*p*-Value)	Unweighted UniFrac (*p*-Value)
**BSp and control groups**	0.001	0.001
**Inulin and control groups**	0.001	0.001
**Combinatorial and control groups**	0.001	0.003

## Data Availability

The original contributions presented in this study are included in the article. Further inquiries can be directed to the corresponding author.

## Supplementary Materials

The following supporting information can be downloaded at https://www.dropbox.com/scl/fo/j6fawoehqk2enho263lid/h?rlkey=aozpp4oaac8h9k0s3nejkglca&st=bnt52z6d&dl=0 (accessed on 8 June 2025).
